# Research of Malting Procedures for Winter Hard Wheat Varieties—Part II

**DOI:** 10.3390/foods10010147

**Published:** 2021-01-12

**Authors:** Vinko Krstanović, Kristina Habschied, Krešimir Mastanjević

**Affiliations:** Faculty of Food Technology, Josip Juraj Strossmayer University of Osijek, 31000 Osijek, Croatia; vkrstano@ptfos.hr (V.K.); kmastanj@gmail.com (K.M.)

**Keywords:** moderately intensive malting procedure, moderately restrictive malting procedure, wheat malt quality

## Abstract

This paper examines the influence of malting process parameters on the wheat malt quality obtained from the assortment of winter red wheat. For this assortment, previous research established that strongly restrictive and strongly intensive malting processes are not suitable, that is, they do not significantly improve the quality of the obtained wheat malts, and in some segments, they even disturb it. Therefore, modifications were introduced to both procedures, and malting was performed with moderately intensive procedure D and moderately restrictive procedure E. Starting wheat, indicators of micromalting process success, and finished wheat malts were analyzed. The results showed that the moderately restrictive malting process (E) significantly improves not only the values for soluble N for almost all tested varieties, but also the values of cytolytic degradation success (wort viscosity, filtration time), and extract yield. The moderately intensive procedure did not improve the determined indicators; for many varieties, the modification even resulted in poorer values. Furthermore, the moderately restrictive procedure allows a strong individual response of a particular variety to the process conditions during malting, which is very important for the assessment of the malting potential for a particular variety. Namely, when assessing the actual malting quality of an individual variety, it is necessary to include amylolytic indicators and indicators of enzymatic strength. In this way, a group of varieties were established which had an increased initial share of total N (varieties no. 7, 8, 9, 10, 12, 13, and 16). These varieties, by this procedure, gave the best quality wheat malts in the entire examined assortment.

## 1. Introduction

European winter wheat varieties have lower protein levels than the spring varieties [1]. The optimization of the process conditions during malting in order to aid and improve the proteolytic, cytolytic, and amylolytic malt quality parameters is very difficult to achieve [2]. Therefore, the optimization of these process conditions can be set only for a certain group of quality indicators. If the wheat of the 2nd malt quality group is characterized by an increased content of total and soluble N, as well as good values for viscosity, then the process conditions that would improve the quality of the finished wheat malt should be set to control excessive proteolysis without significant deterioration of cytolytic and amylolytic indicators. Thus, the malting procedure that would improve the value of the proteolysis indicator should be a procedure with a gradual increase in germination temperature and a decrease in grain moisture [2]. The most extensive research on this topic was conducted by Sacher on soft wheat [3], but he used this procedure only to compare its effect on proteolysis parameters compared to methods with a lower germination temperature, which he considered most acceptable for the assortment he researched. In the first season of this research (Part I) [4], the influence of the malting process on the possibility of improving the quality of finished wheat malts for hard, red wheat from the 2nd wheat malt quality group was established by applying a strongly restrictive and strongly intensive malting procedure, with a standard Middle European Brewing Analysis Commission (MEBAK) procedure as a reference. When malting with a strongly restrictive procedure, poor results were obtained for the values of the proteolysis performance parameters (soluble N, FAN), with additional disturbance and values of cytolytic degradation indicators (viscosity and filtration time, F/C difference) and extract yield [4]. Malting with strongly intensive procedure C also did not improve the values for the wheat malt quality parameters compared to the standard procedure A. Therefore, in the second research season (described in this paper), the modification of intensive and restrictive procedure was carried out in such a way that process conditions were mitigated and re-performed. The test results considered in this paper were obtained from three malting procedures (standard A, moderately intense D, and moderately restrictive E). The intention was to determine whether the modification of the restrictive procedure (B) and the intensive procedure (C) can provide a significant improvement in many indicators of the quality of the finished wheat malt, thus having a consequential impact on the quality of wheat malt and beer.

## 2. Materials and Methods

Sixteen wheat varieties (1-Marija; 2-Liberta; 3-Nina; 4-Adriana; 5-Lana; 6-Ema; 7-Lucija; 8-Ana; 9-Srpanjka; 10-Žitarka; 11-Superžitarka; 12-Barbara, 13-Panonka; 14-OS376-99; 15-OS51-98; and 16-Contur) designated as *Triticum aestivum* L. (ssp. *vulgare*) red grain var. *erythrospermum* or var. *lutescens* were selected for the second season of this research. The preliminary research established that in the examined assortment there are no varieties that meet the criteria of the 1st wheat malt quality group [4,5,6,7,8], but that almost all varieties that showed satisfactory wheat malt performance according to the standard MEBAK micromalting procedure belonged to the 2nd wheat malt quality group. For this research, wheat samples were collected, subjected to the micromalting procedure and then analyzed according to the Analytica-European Brewery Convention (EBC) [9] and Middle European Brewing Analysis Commission (MEBAK) [10] methods, mentioned in Table 1. Total and soluble pentosanes were determined according to Shogren et al. [11]. All analyses were done in duplicate.

Micromalting was carried out in a micro malting plant (Joe White Malting Systems Pty Limited, East Melbourne, Victoria, Australia) using an Automatic Micro Malt Unit, according to the scheme shown in Table 2. Procedure A was the standard MEBAK procedure (Method 2.5.3.1) with the correction of air humidity during the dry steeping phase (85%). Procedure D was moderately restrictive, involving decreasing germination temperatures while procedure E was moderately restrictive and included increasing germination temperatures.

Data analysis: differences between the average values of the raw material, micromalting process indicators, and finished wheat malt quality indicators were analyzed using the analysis of variance (ANOVA) and Fisher’s least significant difference test (LSD), with a statistical significance set at *p* < 0.05. Statistical analysis was carried out using Statistica 13.1. (TIBCO Software Inc., Palo Alto, CA, USA).

## 3. Results

If we compare the results for the initial general quality of wheat varieties (Table 3) with the previous season described in Part I [4], it can be noticed that there was an increase in the share of total N in the grain in almost all varieties (in some significantly). As these are the same varietal experiments as those conducted in the previous season, it is clear that the environmental factor or season has the greatest impact on nitrogen and its fractions in the grain [4,12,13,14]. According to [15] the total soluble N share in the grain is greatly influenced by environmental factors. For winter wheat varieties, Psota et al. [15] determined the percentage of different factors that affect the total soluble N share in grains: factor “year” affects it by 61.7%, factor “location” by 14.4%, and factor “variety” by 11.3%. This increase in total N should consequently be reflected in an increase in soluble N in wheat malt, as well as several other quality indicators related to proteolysis (wheat malt color, FAN, VZ45°). Other examined indicators had approximately the same values as in previous seasons and were in the recommended values for wheat malt production [3,16].

If we first compare the amount and structure of losses between the two examined seasons shown in Table 4, it is immediately noticeable that there was a significant increase in total losses in all malting procedures in this season. This especially refers to standard malting procedure A, which was the same in both seasons. The mean values of losses in procedure A are significantly higher when compared to the previous season, as well as the other two comparative malting procedures D and E. As all initial parameters of raw material quality had similar values as the previous season except for the total N, it is interesting that this increase in losses in all malting procedures is accompanied by an increased content of total N in the examined season. During malting, grain proteins ared hydrolyzed in varying degrees to amino acids [17]. As a result, they become water-soluble, with wheat having a higher proportion of high molecular weight proteins than barley [18], and thus more substrates for proteolysis reactions. Protein hydrolysis also facilitates other hydrolysis reactions that we colloquially call cytolysis and amylolysis. Individual values for the amount and structure of losses by the individual variety and malting process are given in Table 5, Table 6 and Table 7.

In procedure D, the largest stretching of the results was observed for individual cultivars, while the values for procedure E were closely grouped around the median. This is unsurprising because intensive procedures encourage the individual response of each variety (procedure C from the previous season) [4]. In the structure of dry matter losses, it is interesting that similar ratios were obtained in all three procedures, 33–40% were losses on respiration, and the rest were on the germ, with process D having the lowest losses on respiration. In the previous season, a very large difference in the structure of losses was found between the restrictive (B) and the intensive procedure (C), which showed the highest losses on respiration by far. Moderately intensive procedure D follows this trend. Germ growth losses were highest in process A and significantly higher than for process D, which was expected to have the highest value. Process D showed the highest values for swelling capacity, which is interestingly not accompanied by the highest dry matter losses (process A has the largest losses). When comparing the values of the malting process performance indicators, it is noticed that the moderately restrictive procedure E and the strongly restrictive procedure B from the previous season differ significantly, whereby procedure E improves the values for swelling capacity, as well as the amount and structure of losses. This is, in comparison to procedure B in the previous study and procedures A and D in this study. Although the lowest swelling capacity was obtained by procedure E (in comparison to the other two—especially D), it was still higher than the swelling capacity established by the intensive procedure C, which had the highest value in the previous research season.

Mean values for quality indicators of finished wheat malt are given in Table 8. It is interesting that the moderately restrictive process E yielded the lowest values for 1000 grain weight, while the strongly restrictive process B from last season yielded the highest values confirmed by the malting process performance indicators. In this season of research, a significant increase in the concentration of total the N in wheat malt was found (as a consequence of the increase in total N in the starting grain) with relatively small oscillations of in the value of total N in malting procedures within the season. The solubility of malt proteins strongly affects the quality of the beer, including its nutritional value [19]. In terms of values for soluble N both standard A and moderately intensive process D act similarly, while moderately restrictive procedure E resulted in lower values for all varieties. Process E gave relatively satisfactory results for soluble N (787 mg/L). Namely, to conclude that a certain malting process significantly improved this value, soluble N should be <780 mg/L [16], or 600–800 mg/100 g [13]. Most varieties had a value lower than this in malting process E (varieties 6, 7, 8, 10, 13, 14 and 15) with three having limit values (11, 12, and 16). Malting process D resulted in a certain number of cultivars that had a satisfactory value (cultivars 9, 12, 13, 14, 15 and cultivar 16, which had a limit value), while malting process A resulted in the worst results for the value of soluble N (Table 8, Table 9 and Table 10). This lower share of soluble N relative to the starting N in wheat malt can be explained either by lower protease activity (in the case where FAN values are below the recommended 16% in total soluble N [20] or below 120–150 mg/100 g [17]), or that in some cultivars a certain degree of saturation with soluble products of protein degradation occurs during compaction, which results in a certain inhibition of proteolysis [3]. As the concentration of FAN in procedure E for any variety was not below the minimum of 120 mg/100 g, it can be assumed that the cause of this is certain retardation in proteolysis as stated by Sacher [3]; a certain saturation with soluble products of protein degradation. The results of the analysis of the finished wheat malts are given in Table 8, Table 9, Table 10 and Table 11. The results of the analysis of the finished malts are given in Table 8, Table 9, Table 10 and Table 11.

In other indicators of proteolysis success, it can be seen that the values for FAN and Kolbach Index follow the values for total and soluble N (Table 8). In procedure E this is not the case, which is somewhat expected given the ratio of total N: soluble N in this malting process. The Kolbach index is a measure of the degree of protein degradation which is influenced more by the initial concentration of total proteins than the concentration of soluble proteins. Furthermore, it decreases with the increasing protein content in grain, while the influence of initial protein concentration on FAN is unclear [21]. The Hartong number (VZ 45°) and wheat malt color are accompanied by values for soluble N, which is expected because these are values related to the success of the proteolysis process. When it comes to viscosity (Table 8), there is a downward trend in values compared to the previous season, with the mean values for all malting processes being below 1.55 mPa×s, while the required value for wort viscosity is ≤1.65 mPa×s [22,23]. The values for viscosity obtained by the moderately restrictive method E were by far the lowest compared to the other two methods; varieties 2, 4, 12 and 13 also showed very low values. Otherwise, the viscosity values for the examined assortment clearly showed that these are wheat that are typical representatives of the 2nd wheat malt quality group. The values for filtration thus followed the values for viscosity. The values for the extract obtained in malting process E were almost the same as those from the standard malting process A and following the recommended values for light wheat malt [24]. However, they were significantly higher than those in the moderately intensive process D. The F/C difference was also almost identical for processes A and E, yet was expected to be significantly lower for process D. A satisfactory limit attenuation was also found for all malting processes. When we summarize all three malting procedures carried out in this season and include the results from the previous research season, we can conclude that it is possible to improve the grain degradation and the quality of the finished malted wheat grain for the assortment (typical European hard red varieties which belong to the 2nd malt quality group) by a moderately restrictive malting process without too much of an increase in soluble N. 

## 4. Conclusions

The malting process can have a strong effect on the quality of the finished wheat malt. When defining the process conditions we must take into account the initial characteristics of a particular variety or batch that should exhibit its best malting properties under the conditions. When we summarize all three malting procedures performed in this season and include the results from the previous research season, we can conclude that moderately restricting the malting process (moderate temperature rise) can improve grain degradation and the quality of the finished malt for this assortment (typical European hard red varieties which belong to the 2nd malt quality group, characterized by the property and not prone to too deep protein degradation) without an excessive increase of soluble N or disturbance of other important quality indicators.

## Figures and Tables

**Table 1:** Used Middle European Brewing Analysis Commission (MEBAK) and Analytica-European Brewery Convention (EBC) methods for the analysis of wheat and malt.

		Method	
	Unit	MEBAK^®^	EBC^®^
Micromalting		2.5.3.1	
1000 grain weight	g d.wt.		3.4/4.4
Moisture	%		3.2/4.2
Fine extract content	% d. wt.	4.1.4.2.2.	
Extract difference	%	4.1.4.2.10	
Saccharification time	min	4.1.4.2.4.	
Filtration time (min)	min	4.1.4.2.5.	
Total N	% d. wt.	4.1.4.5.1.1.	
Soluble N	mg/L		4.9.1
Kolbach index	%		
Hartong number VZ 45 °C	%	4.1.4.11.	
Final attenuation of wort	%		4.11
Wort colour	EBC	4.1.4.2.8.2.	
Viscosity	mPas. 8.6%e	4.1.4.4.2.	
Diastatic power	°WK	4.1.4.6.	
Vitreosity	%	4.1.3.5.1	
FAN	mg/100 g malt dry m.		4.10
pH	-	4.1.4.2.7.	

**Table 2:** The applied micromalting scheme of wheat samples.

Day	Phase	Malting Procedure		
		A	D	E
1st	Immersion steeping	5 h; t = 14.0 °C;		
	Dry steeping	19 h; t = 14.5 °C		
2nd	Immersion steeping	4 h; t = 14.0 °C;		
	Dry steeping	20 h; t = 14.5 °C		
3rd *	Immersion steeping	2 h; t = 14.0 °C;		
4th	Germination: relative humidity of air in each procedure: r.H. = 85%; sampling during germination was performed daily	t = 14.5 °C	18.0 °C	14.0 °C
5th			15.0 °C	14.5 °C
6th			14.5 °C	15.0 °C
7th			14.0 °C	18.0 °C
8th	Kilning: 19 h (after last hour of germination, kilning was employed and lasted for 19 h; wheat malt was degerminated followed with packaging the samples into paper bags; stored for 2 months before the analysis			

* Control of the degree of steeping at the beginning of the third day and every hour of immersion steeping, when it was found that the grain does not tolerate any further soaking under water, the moisture content in malting procedure A, D, E of (A = 44.5%; D = 44.5%; E = 43.5%) was adjusted with sparging in the germination box (1st day of germination).

**Table 3:** Quality characteristics of the used wheat cultivars (harvest 2018).

	Quality Indicator	Variety															
		1	2	3	4	5	6	7	8	9	10	11	12	13	14	15	16
1	Moisture (%)	11 bc	10.68 cd	10.6 cde	10.74 cd	10.76 cd	10.24 def	11.76 a	11.72 a	11.53 ab	9.73 f	10.58 cde	10 ef	11.52 ab	10.66 cd	11.8 a	10.77 c
2	1000 grain weight (g)	43.3 ef	57.7 a	51.4 b	44 de	45.9 d	42.5 f	42.7 f	39.3 g	40 g	45.8 d	49.9 bc	49.4 c	42 f	42.3 f	43.4 ef	42.4 f
3	Total N (% d.m.)	2.07 b	2.16 b	2.03 a	2.5 b	2.17 b	2.3 b	2.35 b	2.17 b	2.37 b	2.43 b	2.43 b	2.45 b	2.16 b	2.19 b	2.38 b	2.44 b
4	Total proteins (% d.m.)	11.8 ef	12.31 de	11.57 f	14.25 a	12.37 d	13.22 c	13.4 bc	12.37 d	13.51 bc	13.85 ab	13.25 c	13.93 ab	12.31 de	12.48 d	13.57 bc	13.67 bc
5	NIR-HD grain hardness	60 g	65 cde	61 fg	54 h	59 g	63 ef	65 cde	64 de	63 ef	70 a	67 bc	68 ab	66 bcd	68 ab	65 cde	55 h
6	Total pentosans (%d.m.)	7.97 a	7.21 bc	6.62 ef	7.39 b	6.54 efg	7.24 bc	6.43 fg	7.23 bc	6.29 g	7.22 bc	7.18 bc	7.03 cd	7.13 bc	7.31 bc	6.79 de	6.77 de
7	Soluble pentosans (%d.m.)	0.77 bc	0.91 a	0.8 b	0.78 bc	0.79 bc	0.71 f	0.62 f	0.66 def	0.6 f	0.59 f	0.75 bcd	0.66 f	0.77 bc	0.75 bcd	0.75 bcd	0.6 f
8	Total/Soluble pent. (%)	9.7 g	12.6 a	12.1 b	10.6 de	10.6 de	10.4 ef	9.5 gh	9.3 h	9.5 gh	8.3 j	10.4 ef	9.6 g	10.8 cd	10.3 f	10.9 c	8.9 i
9	Vitreosity (%)	30 d	54 a	18 h	18 h	6 k	20 gh	22 fg	15 i	10 j	32 d	30 d	48 b	38 c	26 e	24 ef	22 fg

Values are means of two measurements. Values displayed in the same lines and tagged with different letters are significantly different (*p* < 0.05).

**Table 4:** Mean values of the testing varieties for the quality parameters of the micromalting process by season and malting procedures.

		Malting Procedures			
		A	D	E	Recommended Value
1.	moisture after 48 h (%)	43.31 a ± 0.56	46.48 a ± 0.55	41.43 a ± 0.40	>40%
2.	respiration losses (% g/dm)	3.76 d ± 0.30	3.34 d ± 0.25	3.51 d ± 0.26	-
3.	germination losses % g/dm)	7.07 c ± 0.60	5.8 c ± 0.40	5.12 c ± 0.32	-
4.	total losses (% g/dm)	11.21 b ± 0.41	9.11 b ± 0.58	8.56 b ± 0.22	<10%

Values are means of two measurements ± standard deviation. Values displayed in the same lines and tagged with different letters are significantly different (*p* < 0.05).

**Table 5:** Results of micromalting analysis (procedure A).

	Quality Indicator	Variety															
		1	2	3	4	5	6	7	8	9	10	11	12	13	14	15	16
1.	moisture after 48 h (%)	42.1 fg	42.3 ef	42.4 ef	42.6 e	41.7 g	41.8 g	42.1 fg	42.1 fg	44.4 b	44.5 b	43.2 d	43.9 c	44.4 b	45.1 a	44.6 b	44.8 ab
2.	respiration losses (% g/dm)	3.5 f	3.9 e	3.8 e	4.53 d	5.2 b	4.8 c	2.2 j	3.5 f	3.0 h	5.4 a	3.6 f	5.2 b	2.7 i	3.3 g	2.7 i	2.8 i
3.	germination losses % g/dm)	7.5 d	5.9 h	6.9 g	5.9 h	5.3 j	5.5 i	9.5 b	7.6 d	7.3 e	9.9 a	8.0 c	7.1 f	8.1 c	8.1 c	4.6 k	5.9 h
4.	total losses (% g/dm)	11.0 g	9.8 j	10.7 h	10.4 i	13.5 b	13.3 c	11.7 e	11.1 g	10.3 i	15.3 a	11.6 e	12.3 d	10.8 h	11.4 f	7.30 l	8.80 k

Values in Table 4 are means of two measurements. Values displayed in the same lines and tagged with different letters are significantly different (*p* < 0.05).

**Table 6:** Results of micromalting analysis (procedure D).

	Quality Indicator	Variety															
		1	2	3	4	5	6	7	8	9	10	11	12	13	14	15	16
1.	moisture after 48 h (%)	49.3 c	46.9 e	47.7 de	50.5 b	48.6 cd	47.8 de	51.3 ab	52.0 a	43.7 fgh	43.4 fghi	43.0 hi	42.5 i	43.7 fgh	44.4 fg	43.3 ghi	44.5 f
2.	respiration loss. (% g/dm)	3.1 fg	3.1 fg	3.0 gh	2.1 j	3.6 d	3.7 d	3.1 fg	4.6 b	3.0 gh	5.3 a	4.2 c	3.3 e	3.2 ef	2.9 h	3.0 gh	2.5 i
3.	germination loss. % g/dm)	11.7 a	4.7 h	3.8 k	5.2 g	3.9 k	7.4 d	8.1 c	11.0 b	4.3 i	6.4 e	5.8 f	5.3 g	4.1 j	4.2 ij	3.2 l	3.3 l
4.	total losses (% g/dm)	14.8 b	7.7 b	6.8 b	7.2 b	7.5 b	11.1 b	11.2 b	15.6 b	7.3 b	11.7 b	10.0 b	8.6 b	7.2 b	7.0 b	6.2 b	5.8 b

Values in Table 5 are means of two measurements. Values displayed in the same lines and tagged with different letters are significantly different (*p* < 0.05).

**Table 7:** Results of micromalting analysis (procedure E).

	Quality Indicator	Variety															
		1	2	3	4	5	6	7	8	9	10	11	12	13	14	15	16
1.	moisture after 48 h (%)	42.7 a	41.8 d	42.2 c	42.2 c	41.8 d	41.6 e	41.5 e	42.5 b	41.1 f	41.0 f	40.3 ij	40.2 j	40.5 gh	40.4 hi	40.5 g	42.4 b
2.	respiration losses (% g/dm)	3.4 e	4.0 cd	3.9 d	2.7 g	2.4 h	2.3 h	3.2 f	4.1 c	4.7 a	4.4 b	3.5 e	3.96 cd	3.5 e	4.0 cd	3.4 e	2.7 g
3.	germination losses (% g/dm)	5.4 de	4.2 g	5.4 de	6.35 a	5.7 c	6.5 a	5.2 e	4.1 g	4.3 fg	6.0 b	4.5 f	5.7 c	5.6 cd	4.3 fg	4.2 g	4.2 g
4.	total losses (% g/dm)	8.8 bc	8.2 cdef	9.3 b	9.2 b	8.1 def	8.8 bc	8.3 cde	8.1 def	8.0 ef	10.4 a	8.2 cdef	8.7 bcd	9.1 b	8.3 cde	7.6 f	6.9 g

Values in Table 6 are means of two measurements. Values displayed in the same lines and tagged with different letters are significantly different (*p* < 0.05).

**Table 8:** Mean values of the testing varieties for the quality parameters of wheat malt.

		Recommended Values	Malting Procedures		
			A	D	E
1.	1000 grain weight (g d.wt.)	-	35.9 ef ± 0.55	36.7 e ± 0.30	34.7 ef ± 3.97
2.	Vitreosity (%)	5–10 ****	9 fg ± 6.5	9 f ± 7.17	13 fg ± 6.88
3.	Total N (% d.wt.)	>1.8 ***	2.2 g ± 0.19	2.15 f ± 0.29	2.25 g ± 0.16
4.	Soluble N (mg/L)	700–900 *	897 a ± 140.42	835 a ± 101.61	787 a ± 146.67
5.	Kolbach Index (%)	<42 ****	40.7 e ± 6.58	38.3 e ± 6.87	35.5 ef ± 7.77
6.	FAN (mg/100 g dry wt.)	80–110 *	142 c ± 69.33	135 c ± 9.77	132 c ± 7.05
7.	Fine extract content (% d.wt.)	-	83.89 d ± 1.26	80.6 d ± 12.39	83.43 d ± 1.62
8.	Extract difference (%)	<2.5 ****	1.37 g ± 0.92	1.1 f ± 0.46	1.45 g ± 0.68
9.	Wort colour (EBC u.)	3–5 **	5.2 g ± 0.71	4.7 f ± 0.86	4.5 g ± 0.77
10.	Filtration time (min)	<60 **	48 e ± 11.53	64 d ± 24.39	43 e ± 8.16
11.	pH	5.9–6.1	6.09 g ± 0.08	6.1 f ± 0.06	6.1 g ± 0.07
12.	Viscosity (mPas. 8.6%e)	<1.8	1.476 g ± 0.06	1.550 f ± 0.08	1.418 g ± 0.03
13.	Hartong number VZ 45 °C (%)	>33 **	37.8 e ± 5.25	35.5 e ± 4.95	34.7 ef ± 6.03
14.	Diastatic power WK°	250–420	253 b ± 5.83	264 b ± 6.31	267 b ± 6.12
15.	Final attenuation of wort (%)	≈78 **	82.9 d ± 0.41	84.4 d ± 0.94	83.8 d ± 0.41

Values are means of two measurements ± standard deviation. Values displayed in the same lines and tagged with different letters are significantly different (*p* < 0.05). Recommended values are from different literature references * [3], ** [10], *** [12], **** [20].

**Table 9:** Results of wheat malt analysis (procedure A).

	Quality Indicator	Variety															
		1	2	3	4	5	6	7	8	9	10	11	12	13	14	15	16
1	Moisture (%)	4.57 de	4.15 g	4.40 fg	3.91 i	5.01 b	5.02 b	6.48 a	4.82 c	4.65 d	4.54 de	5.03 b	5.01 b	4.62 de	4.52 ef	4.63 de	4.34 fg
2	1000 grain weight (g d.wt.)	34.6 g	44.4 a	41.8 b	36.2 e	35.9 f	33.4 i	34.0 h	30.2 l	31.6 k	39.2 d	39.0 d	40.0 c	33.4 i	34.0 h	32.6 j	34.6 g
3	Vitreosity (%)	4.0 e	24.0 a	4.0 e	6.0 de	6.0 de	10.0 cde	16.0 bc	4.0 e	4.0 e	16.0 bc	12.0 cd	22.0 ab	10.0 cde	4.0 e	6.0 de	4.0 e
4	Total N (% d.wt.)	1.9 e	2.1 cde	1.9 e	2.5 a	2.1 cde	2.3 abc	2.46 a	2.2 bcd	2.3 abc	2.3 abc	2.3 abc	2.4 ab	2.0 de	2.0 de	2.13 cde	2.3 abc
5	Soluble N (mg/L)	899.0 f	1214.6 b	968.0 d	987.0 c	1219.0 a	795.0 ij	797.0 i	836.0 h	778.0 jk	900.0 f	919.0 e	900.0 f	762.0 k	732.0 l	788.0 ij	856.0 g
6	Kolbach Index (%)	45.9 d	49.2 c	51.0 b	40.0 e	57.0 a	35.1 ij	35.5 ij	38.8 efg	34.1 j	39.8 e	39.4 ef	38.6 efg	37.3 gh	35.9 hi	36.0 hi	37.9 fg
7	FAN (mg/100 g dry wt.)	135.4 abc	132.2 bcd	125.7 cde	124.0 def	118.5 efgh	111.5 ghi	115.7 efghi	107.2 i	110.6 hi	138.8 ab	138.8 ab	144.1 a	118.7 efghi	110.3 hi	122.2 defg	114.3 fghi
8	Fine extract content (% d.wt.)	83.8 cde	83.5 de	86.3 a	82.3 g	87.0 a	85.0 b	83.6 de	83.8 cde	83.2 def	83.9 cd	82.6 fg	83.5 de	84.5 bc	83.0 efg	83.9 cd	82.3 g
9	Extract difference (%)	0.2 j	0.3 ij	0.4 i	0.6 h	4.1 a	1.2 f	1.5 de	1.6 d	1.5 de	1.0 g	1.8 c	1.2 f	1.8 c	1.2 f	1.4 e	2.1 b
10	Saccharification time (min)	<10	<10	<10	<10	<10	<10	<10	<10	<10	<10	<10	<10	<10	<10	<10	<10
11	Wort colour (EBC u.)	5.6 b	5.7 b	4.9 e	4.5 g	6.5 a	5.0 de	4.7 f	4.1 h	5.0 de	5.1 d	5.4 c	5.4 c	5.0 de	4.1 h	4.9 e	6.6 a
12	Filtration time (min)	45 e	40 f	65 b	40 f	60 c	75 a	50 d	45 e	50 d	45 j	45 e	40 f	60 c	40 f	40 f	30 g
13	pH	6.1 b	6.0 c	6.1 b	6.0 c	6.1 b	6.2 a	6.1 b	6.1 b	6.1 b	5.9 d	6.1 b	6.1 b	6.2 a	6.1 b	6.1 b	6.2 a
14	Viscosity (mPas. 8.6%e)	1.493 de	1.479 cde	1.573 b	1.5123 c	1.615 a	1.506 cd	1.474 fg	1.486 ef	1.457 hi	1.461 gh	1.441 i	1.406 j	1.366 k	1.468 gh	1.413 j	1.459 gh
15	Hartong number VZ 45 °C (%)	43.3 b	44.6 b	35.8 def	35.2 def	51.5 a	36.6 de	39.6 c	34.7 f	31.8 g	36.1 def	40.4 c	40.4 c	34.7 ef	34.5 f	36.7 d	30.3 g
16	Diastatic power (°WK)	245 c	256 abc	253 abc	255 abc	251 abc	254 abc	250 abc	254 abc	249 abc	263.6 ab	245 c	266 a	255 abc	248 bc	246 c	255 abc
17	Final attenuation of wort (%)	83.4 bc	82.5 cde	83.6 bc	84.2 ab	81.9 def	83.1 bcd	82.4 cdef	82.9 bcde	83.4 bc	82.3 cdef	82.7 bcde	81.2 f	81.6 ef	85.4 a	83.0 bcd	82.9 cdef

Values are means of two measurements. Values displayed in the same lines and tagged with different letters are significantly different (*p* < 0.05).

**Table 10:** Results of wheat malt analysis (procedure D).

	Quality Indicator	Variety															
		1	2	3	4	5	6	7	8	9	10	11	12	13	14	15	16
1	Moisture (%)	5.18 a	4.61 d	4.87 c	4.51 e	4.91 bc	4.97 b	4.5 ef	4.47 efg	5.21 a	4.88 bc	4.14 h	4.4 g	5.15 a	4.41 fg	4.96 bc	4.54 de
2	1000 grain weight (g d.wt.)	34.0 gh	46.5 a	42.8 b	36.9 de	37.4 d	33.1 gh	33.5 gh	31.4 i	32.9 h	40.8 c	39.4 c	39.9 c	36 de	35.8 ef	34.4 fg	34.4 fg
3	Vitreosity (%)	6 de	24 a	2 e	4 de	2 e	8 cd	16 b	4 de	4 de	16 b	14 b	22 a	12 bc	4 de	8 cd	2 e
4	Total N (% d.wt.)	1.86 g	2.1 de	1.97 fg	2.4 ab	2.17 de	2.22 cd	1.2 h	2.17 de	2.3 bc	2.3 bc	2.43 a	2.42 ab	2.15 de	2.08 ef	2.22 cd	2.47 a
5	Soluble N (mg/L)	856 cde	945 bc	873 cde	917 bcd	966 abc	829 de	911 bcd	884 cde	660 h	865 cde	861 cde	669.3 h	689 gh	697 gh	736 fgh	793 efg
6	Kolbach Index (%)	55.7 a	48.6 b	42.7 c	38.2 e	43.2 c	37.2 ef	40.5 d	40.6 d	28.7 i	37.4 ef	35.5 fg	34.6 g	31.8 h	33.6 gh	31.7 h	33.5 gh
7	FAN (mg/100 g dry wt.)	146.2 ab	144.2 abc	136.4 bcd	143.0 abc	128.1 de	120.4 e	131.1 cde	119.1 e	129.4 de	148.2 ab	146.5 ab	151.7 a	131.1 cde	138.2 bcd	137.1 bcd	135.4 bcd
8	Fine extract content (% d.wt.)	0.6 f	0.8 e	0.5 fg	1.3 c	1.1 d	1.2 cd	1.1 d	0.6 f	2.2 a	1.1 d	1.1 d	1.3 c	1.5 b	0.8 e	0.4 g	1.6 b
9	Extract difference (%)	0.6 f	0.8 e	0.5 fg	1.3 c	1.1 d	1.2 cd	1.1 d	0.6 f	2.2 a	1.1 d	1.1 d	1.3 c	1.5 b	0.8 e	0.4 g	1.6 b
10	Saccharification time (min)	<10	15–20	<10	10–15	10–15	<10	<10	<10	<10	<10	<10	<10	10–15	<10	<10	10–15
11	Wort colour (EBC u.)	6.1 b	5.2 cd	3.3 j	6.3 a	4.6 g	4.8 ef	4.9 e	4.1 h	4.7 fg	5.1 d	5.1 d	5.2 cd	4.0 hi	3.3 j	3.9 i	5.3 c
12	Filtration time (min)	45 g	50 f	90 c	80 d	120 a	50 f	80 d	110 b	60 e	44.6 g	50 f	40 h	60 e	60 e	40 h	45 g
13	pH	6.0 c	6.0 c	6.1 b	6.1 b	6.1 b	6.0 c	6.1 b	6.2 a	6.1 b	6.1 b	6.1 b	6.1 b	6.2 a	6.1 b	6.1 b	6.2 a
14	Viscosity (mPas. 8.6%e)	1.507 ghi	1.6020 c	1.711 b	1.561 de	1.7410 a	1.531 fg	1.485 ij	1.5467 ef	1.467 j	1.488 ij	1.524 fgh	1.488 ij	1.496 i	1.585 cd	1.543 ef	1.503 hi
15	Hartong number VZ 45 °C (%)	50.7 a	39.2 cd	29.5 hi	28.8 i	33.3 f	41.0 c	43.9 b	40.2 c	31.0 gh	37.6 de	36.4 e	35.8 e	29.5 hi	31.3 fgh	32.3 fg	27.8 i
16	Diastatic power (°WK)	261 abcd	263 abcd	268 abc	261 abcd	268 abc	255 bcd	259 abcd	252 cd	261 abcd	273 a	258 abcd	274 a	248 d	261 abcd	270 ab	266 abc
17	Final attenuation of wort (%)	84.0 efg	83.3 gh	83.6 fgh	85.1 b	84.9 bcd	84.8 bcde	84.1 cdefg	83.3 gh	84.6 bcde	83.0 h	83.6 fgh	84.2 cdef	83.5 fgh	86.5 a	85.0 bc	84.1 defg

Values are means of two measurements. Values displayed in the same lines and tagged with different letters are significantly different (*p* < 0.05).

**Table 11:** Results of wheat malt analysis (procedure E).

	Quality Indicator	Variety															
		1	2	3	4	5	6	7	8	9	10	11	12	13	14	15	16
1	Moisture (%)	6.44 c	6.43 c	6.55 bc	6.22 d	5.72 f	5.87 e	5.99 e	5.87 e	5.91 e	5.70 f	5.38 g	5.59 f	6.69 a	6.23 d	6.66 ab	6.24 d
2	1000 grain weig. (g d.wt.)	33.6 cde	44.5 a	40.3 ab	34.9 bcde	34.0 bcde	32.2 de	32.6 cde	20.8 f	30.2 e	37.5 bcd	36.8 bcd	38.9 abc	33.0 cde	32.9 cde	31.2 de	33.1 cde
3	Vitreosity (%)	8 fgh	30 a	6 gh	12 def	4 h	10 efg	14 de	8 fgh	8 fgh	16 cd	14 de	22 b	20 bc	10 efg	16 cd	6 gh
4	Total N (% d.wt.)	2.0 c	2.1 bc	2.0 c	2.5 a	2.1 bc	2.2 abc	2.2 abc	2.3 abc	2.3 abc	2.3 abc	2.5 a	2.5 a	2.2 abc	2.16 bc	2.4 ab	2.4 ab
5	Soluble N (mg/L)	850.0 e	989.0 b	900.0 d	937.0 c	1141.0 a	707.0 h	701.0 h	667.0 i	803.0 f	626.0 i	802.0 f	779.0 g	626.0 j	608.0 k	712.0 h	807.0 f
6	Kolbach Index (%)	42.4 c	46.9 b	45.9 b	37.4 d	54.0 a	32.3 fg	31.7 g	29.4 ij	34.5 e	27.3 k	33.3 ef	31.3 gh	28.4 jk	29.3 ij	30.1 hi	33.9 e
7	FAN (mg/100 g dry wt.)	136.2 cd	134.3 de	129.2 f	138.0 c	132.5 e	132.4 e	137.8 c	118.2 i	121.6 h	135.7 d	140.1 b	144.9 a	125.1 g	125.3 g	130.3 f	130.3 f
8	Fine extract cont. (%d.wt.)	81.8 f	86.0 a	83.9 cd	81.8 f	86.3 a	82.8 def	85.2 ab	82.9 def	82.6 ef	80.3 g	82.0 f	84.4 bc	84.4 bc	82.6 ef	83.6 cde	81.9 f
9	Extract difference (%)	0.8 i	1.8 de	2.5 a	1.9 cd	0.9 hi	2.0 c	1.4 f	0.9 hi	1.2 g	2.2 b	1.0 h	0.4 j	1.4 f	2.3 b	1.7 e	0.2 k
10	Saccharif. time (min)	<10	<10	<10	10-15	<10	10-15	<10	<10	<10	10-15	<10	<10	10-15	<10	<10	10-15
11	Wort colour (EBC u.)	5.8 a	5.4 b	4.2 f	4.8 d	5.3 b	5.0 c	4.0 g	3.5 h	4.0 g	4.0 g	4.7 d	4.5 e	4.0 g	3.2 i	4.0 g	5.7 a
12	Filtration time (min)	55 a	50 b	50 b	35 e	45 c	30 f	40 d	40 d	55 a	35 e	30 f	45 c	50 b	45 c	40 d	35 e
13	pH	6.2 a	6.0	6.1 b	6.1 b	6.0 c	6.2 a	6.2 a	6.2 a	6.1 b	6.2 a	6.1 b	6.1 b	6.2 a	6.2 a	6.2 a	6.2 a
14	Viscosity (mPas. 8.6%e)	1.444 bc	1.365 i	1.429 cde	1.392 h	1.431 cde	1.402 gh	1.408 fgh	1.459 ab	1.421 ef	1.419 ef	1.439 cd	1.408 fgh	1.359 i	1.470 a	1.424 def	1.411 fgh
15	Hartong num. VZ 45 °C (%)	41.8 b	41.5 b	37.9 c	35.3 de	49.9 a	28.1 ij	31.7 fg	33.5 fg	35.4 d	29.9 ghi	35.8 d	36.4 cd	29.8 hi	28.6 ij	31.26 gh	27.76 j
16	Diastatic power (°WK)	260 hi	258 i	269 cde	258 i	277 a	265 efg	267 def	262 ghi	265 efg	264 efg	268 cdef	270 cd	271 bcd	272 bc	275 ab	265 efg
17	Final attenuat. of wort (%)	83.4 ef	84.2 abc	84.0 abcd	83.7 cdef	83.8 cdef	84.1 abcd	83.9 bcde	83.4 ef	84.1 abcd	83.5 def	84.6 a	83.4 ef	83.6 def	84.5 ab	84.1 abcd	83.2 f

Values are means of two measurements. Values displayed in the same lines and tagged with different letters are significantly different (*p* < 0.05).

## Data Availability

Restrictions apply to the availability of these data. Data was obtained from the Agricultural Institute Osijek and are available [from the authors] with the permission of the Agricultural Institute Osijek.
